# 

**Published:** 1904-10

**Authors:** 


### BOOKS RECEIVED.

A Textbook of Diseases of Women. By Charles B. Penrose, M.D., Ph.D., formerly Professor of Gynecology in the University of Pennsylvania. Fifth edition, thoroughly revised. Octavo volume of 539 pages, with 221 fine original illustrations. Philadelphia, New York, London: W. B. Saunders & Company. 1904. (Cloth, $3.75 net.)

A Textbook of Materia Medica: Including Laboratory Exercises in the Histologic and Chemic Examinations of Drugs. For Pharmaceutic and Medical Schools, and for Home Study. By Robert A. Hatcher, Ph.G., M.D., Instructor in Pharmacology in Cornell University Medical School of New York City; and Torald Sollmann, M.D., Assistant Professor in Pharmacology and Materia Medica in the Medical Department of the Western Reserve University of Cleveland. Duodecimo volume of about 400 pages, illustrated. Philadelphia, New York, London: W. B. Saunders & Co. 1904. (Flexible leather, $2.00 net.)

Examination of the Urine. By G. A. deSantos Saxe, M.D., Pathologist to the Columbus Hospital, New York City. Duodecimo volume of 391 pages, fully illustrated, including 8 colored plates. Philadelphia, New York, London: W. B. Saunders & Co. 1904. (Flexible leather, $1.50 net.)

A Handbook of Surgery. For Students and Practitioners. By Frederic R. Griffith, M.D., Surgeon to the Bellevue Dispensary, New York City; Assistant Surgeon at the New York Polyclinic School and Hospital. Duodecimo volume of 579 pages, containing 417 illustrations. Philadelphia, New York, London: W. B. Saunders & Co. 1904. (Flexible leather, $2.00 net.)

A Textbook of Clinical Diagnosis. By Laboratory Methods. For the use of Students, Practitioners, and Laboratory Workers. By L. Napoleon Boston, A.M., M.D., Associate in Medicine and Director of the Clinical Laboratories of the Medico-Chirurgical College, Philadelphia; formerly Bacteriologist at the Philadelphia Hospital and at the Ayer Clinical Laboratory of the Pennsylvania Hospital. Octavo volume of 547 pages, with 320 illustrations, many of them in colors. Philadelphia, New York, London: W. B. Saunders & Co. 1904. (Cloth, $4.00 net; sheep or half morocco, $5.00 net.)

A System of Practical Surgery. By Drs. E. von Bergmann, Berlin, P. von Bruns, Tübingen and J. von Mikulicz, Breslau. Volume IV. Surgery of the Alimentary Tract. Translated and edited by William T. Bull, M. D., Professor of Surgery, College of Physicians and Surgeons, Columbia University, New York. To be complete in five imperial volumes. Philadelphia and New York: Lea Brothers & Company. 1904. (Price, per volume: cloth, $6.00; leather, $7.00; half morocco, $8.50.)

A Practical Treatise on Genitourinary and Venereal Diseases and Syphilis. By Robert W. Taylor, A.M., M.D., Clinical Professor of Genito-urinary Diseases at the College of Physicians and Surgeons (Columbia University) New York. Third edition, thoroughly revised. Octavo, pp. 757. With 163 illustrations and 39 plates in colors and monochrome. New York and Philadelphia: Lea Brothers & Co. 1904. (Price, cloth, $5.00; leather, $6.00; half morocco, $6.50, net.)

The Practice of Obstetrics. Designed for the Use of Students and Practitioners of Medicine. By J. Clifton Edgar, Professor of Obstetrics and Clinical Midwifery in the Cornell University Medical College, New York. Royal octavo, pp. 1153. With 1264 illustrations, including 5 colored plates and 38 figures printed in colors. Second edition, revised. Philadelphia: P. Blakiston's Son & Co. 1904. (Price, $6.00, net.)

Saunders' Question Compends: Essentials of Chemistry, Organic and Inorganic. Containing also questions on Medical Physics, Chemical Philosopy, Medical Processes, Toxicology, etc. By Lawrence Wolff, M.D., formerly Demonstrator of Chemistry at the Jefferson Medical College, Philadelphia. Sixth edition, thoroughly revised. By A. Ferree Witmer, Ph.G., formerly Assistant Demonstrator in Physiology at the University of Pennsylvania. Duodecimo volume of 225 pages, fully illustrated. Philadelphia, New York, London: W. B. Saunders & Co. 1904. (Cloth, $1.00 net.)

Progressive Medicine, Volumne III, September, 1904. A Quarterly Digest of Advances, Discoveries and Improvements in the Medical and Surgical Sciences. Edited by Hobart Amory Hare, M.D., Professor of Therapeutics and Materia Medica in the Jefferson Medical College of Philadelphia. Octavo, 284 pages, 19 illustrations. Philadelphia and New York: Lea Brothers & Co., Publishers. (Per annum, in four cloth-bound volumes, $9.00; in paper binding, $6.00, carriage paid to any address.)

Davis' Obstetrics. A Treatise on Obstetrics. For Students and Practitioners. By Edward P. Davis, A.M., M.D., Professor of Obstetrics in Jefferson Medical College; Professor of Obstetrics and Pediatrics in the Philadelphia Polyclinic, etc. New (second) edition, thoroughly revised and much enlarged. Octavo, 800 pages, with 274 engravings and 39 full-page plates in colors and monochrome. (Cloth, $5.00 net; leather, $6.00 net.)

A Handbook of Pathological Anatomy and Histology with an introductory section on Post-Mortem Examinations and the Methods of Preserving and Examining Diseased Tissues. By Francis Delafield, M.D., LL.D., Emeritus Professor of the Practice of Medicine, College of Physicians and Surgeons, Columbia University; and T. Mitchell Prudden, M.D., LL.D., Professor of Pathology and Director of the Department of Pathology, College of Physicians and Surgeons, Columbia University, New York. Seventh edition, with 13 full-page plates and 545 illustrations in the text, in black and colors. New York: William Wood & Co. 1904.

Kerke's Handbook of Physiology. Revised by Frederick C. Busch. B.S., M.D., Professor of Physiology, Medical Department, University of

Buffalo. Fifth American revision, with 535 illustrations, including many in colors. New York: William Wood & Co. 1904.

Simon's Physiological Chemistry. A Textbook of Physiological Chemistry. For Students and Practitioners of Medicine. By Charles E. Simon, M.D., late Resident Physician, Johns Hopkins Hospital; author of Simon's Clinical Diagnosis, etc. New (second) edition. Revised and enlarged. Octavo, 500 pages. Philadelphia and New York: Lea Brothers & Co., Publishers. (Cloth, $3.25 net.)

Transactions of the Medical Society of the State of New York. Ninety-Eighth Annual Session, January 26, 27, 28, 1904. Frederic C. Curtis, M.D., Secretary. Published by the Society. 1904.

Lea's Series of Medical Epitomes. Nagel's Epitome of Nervous and Mental Diseases. A Manual for Students and Physicians. By Joseph Darwin Nagel, M.D., Consulting Physician to the French Hospital, New York. In one duodecimo volume of 276 pages, with 46 illustrations. Philadelphia and New York: Lea Brothers & Co., Publishers. 1904. (Cloth, $1.00 net.)

Thirtieth Annual Report of the Secretary of the State Board of Health of the State of Michigan, for the fiscal year ending June 30, 1902. Henry B. Baker, M.D., Secretary. By authority, Lansing. Robert Smith & Co., State Printers and Binders. 1903.

Clinical Urinology. By Alfred C. Croftun, Professor of Medicine, Chicago, Post-Graduate Medical College and Hospital; Physician in Chief to Saint Mary's Hospital; Pathologist to Saint Luke's Hospital. Illustrated. New York: William Wood & Co. 1904.

Magee and Johnson's Epitome of Surgery. A Manual for Students and Practitioners. By M. D'Arcy Magee, A.M., M.D., Demonstrator of Surgery and Lecturer on Minor Surgery; and Wallace Johnson, Ph.D., M.D., Demonstrator of Pathology and Bacteriology in Georgetown University Medical School, Washington, D. C. In one duodecimo volume of 295 pages, with 129 engravings. Philadelphia and New York: Lea Brothers & Co., Publishers. 1904. (Cloth, $1.00 net.)

Textbook of Nervous Diseases and Psychiatry. For the use of Students and Practitioners of Medicine. By Charles L. Dana, A.M., M.D., Professor of Nervous Diseases and (ad interim) of Mental Diseases in Cornell University Medical College; Visiting Physician to Bellevue Hospital, etc. Sixth revised and enlarged edition. Illustrated by 244 engravings and three plates in black and colors. New York: William Wood & Co. 1904.

Regional Minor Surgery. By George Gray Van Schaick, Consulting Surgeon to French Hospital, New York. Second edition, enlarged and revised, 228 pages, bound in cloth, profusely illustrated. New York: International Journal of Surgery Co. 1904. (Price, $1.50.)

How to Cook for the Sick and Convalescent. Arranged for the Physician, Trained Nurse, and House Use. By Helena V. Sachse. Second edition, revised and enlarged. Philadelphia: J. B. Lippincott Company. 1904.

Transactions of the Ninth Annual Meeting of the American Laryngological, Rhinological and Otological Society, held at Lexington, Ky., April 30, May 1, and 3, 1903. Wendell C. Phillips, Secretary, New York. Published by the Society. 1903.

University of Pennsylvania. Contributions from the William Pepper Laboratory of Clinical Medicine. (Reprints). No. 4. Philadelphia. 1903.

A Practical Treatise of Diseases of the Skin. For the Use of Students and Practitioners. By James Nevin Hyde, A.M., M.D., Professor of Skin, Genitourinary and Venereal Diseases, Rush Medical College, Chicago;

Dermatologist to the Presbyterian, Augustana, and Michael Rush Hospitals of Chicago, etc.; and Frank Hugh Montgomery, Associate Professor of Skin, Genitourinary and Venereal Diseases, Rush Medical College, Chicago; Professor of Skin and Veneral Diseases, Chicago Clinical School, etc. Seventh and revised edition. Illustrated with 107 engravings and 34 plates in colors and monochromes. Philadelphia and New York: Lea Brothers & Co. 1904.

